# Preliminary study on the prevalence of workplace psychological terror among Hungarian athletes

**DOI:** 10.3389/fspor.2026.1828117

**Published:** 2026-08-07

**Authors:** Viktória Resperger, Tamás Berki, Anikó Kapros

**Affiliations:** 1 Department of Psychology and Sport Psychology, Institute of Economic and Social Sciences, Hungarian University of Sports Science, Budapest, Hungary; 2 Department of Physical Education Theory and Methodology, Hungarian University of Sports Science, Budapest, Hungary; 3 Doctoral School of Military Sciences, Ludovika University of Public Service, Budapest, Hungary

**Keywords:** athlete abuse, athlete safeguarding, HR in sports, mobbing actions, workplace psychological terror

## Abstract

The article primarily examines sport as an atypical workplace, highlighting various forms of workplace psychological terror, also known as workplace mobbing, that can be observed within the field of sport. Examples that can occur in a sports workplace, such as treatment violating human dignity, sexual and physical harassment, discrimination, and emotional manipulation, as well as fear of punishment and coercion into prohibited actions, constitute a significant factor in the lives of athletes worldwide. The primary objective of this research was to assess the prevalence of workplace psychological terror among Hungarian athletes and, based on the findings, formulate scientifically substantiated recommendations for remedial actions. The researchers utilized a self-administered online questionnaire to examine the prevalence of psychological terror among athletes, along with other forms of workplace abuse that occur within the sporting environment. The instrument was created by the research team and evaluated nine categories of athlete mobbing actions. The findings indicated that various types of workplace psychological terror are present in the Hungarian sports system. Based on the results, the authors suggest that, to combat and prevent all forms of workplace psychological terror in sport, systemic change is needed, grounded in an Occupational and Organizational Psychology (OOP) approach. The results of our preliminary study suggest that the questionnaire section examined may be suitable for further methodological development and domestic validation.

## 1 Introduction

Sports are frequently linked with athletes who lead healthy lifestyles, possess aesthetic appeal, participate in community activities, and uphold fair play. However, upon closer inspection, it becomes evident that the domain of sports is not devoid of challenges. The hierarchical nature of sports organizations, the intense pressure to excel, and prevailing community standards can all foster the development of various types of harassment and abuse (1).

It seems that discussions surrounding the hard work, perseverance and numerous sacrifices that underpin sport attract relatively limited attention and the frequency with which athletes endure abuse in various forms appears to receive even less. The fact that all these issues manifest early in the lives of athletes is perplexing, as we tend to regard such experiences as an inevitable aspect of attaining high performance (2). The culture within elite sports, characterized by a tolerance for certain behaviors and significant power imbalances in coach-athlete relationships, can lead to the normalization of potentially abusive practices. This normalization can consequently delay an athlete's recognition of their experiences as abuse as these experiences might initially be perceives as inherent to the demanding nature of hard work and sacrifice in high-level competition (3).

The initial focus of this article was to investigate the types of workplace psychological terror that can be identified within the realm of sports. During our investigation, numerous Hungarian athletes publicly shared the extent and form of abuse they had endured throughout their athletic careers. This issue is not confined to Hungary. Internationally, several Olympians have likewise spoken openly about their experiences of abuse. Well-known examples include Simone Biles, Aly Raisman, and Catherine Lyons, although the list is considerably longer and spans multiple disciplines (1, 4). Public testimonies by former athletes have also contributed to raising awareness of safeguarding in sport. For example, former tennis player Jelena Dokic has openly discussed her experiences of abuse, highlighting the importance of prevention and athlete protection (5).

There are also instances involving high-profile court cases being publicly recognized, such as the case of the former physician of the USA Gymnastics team, Dr. Larry Nassar. The former physician participated in four Olympic Games and, in 2018, was sentenced to 40 to 125 years in prison. He was accused of sexually abusing approximately 256 female athletes throughout his career in sports (6). As the results show, incidents involving various forms of workplace psychological terror have also occurred in Hungary. Notable national cases include the investigation by the Hungarian Swimming Federation against coach György Turi. Following the investigation, an apology was extended to those athletes who had fallen victim to coaching methods, which included physical and psychological abuse (7). Turi has resigned from previous positions at the Federation but is still the head of the swimming department at a national sport club (8).

The starting point of the research was the identification of workplace stressors within the realm of sport, which we interpreted as factors that are decisive in terms of organizational functioning and athlete well-being (9). Our study focuses on a small section of a larger, comprehensive questionnaire-based research project. The part of the questionnaire analyzed in this study has not been presented previously, but results from other sections of the measurement tool have already been published (9). The results of a section of our survey, which dealt with workplace psychological terror, showed marked patterns in several cases, drawing attention to the fact that certain stressors play a prominent role in the workplace experiences of Hungarian athletes.

Although the results of the workplace psychological terror section of our research are preliminary and cannot be considered representative, the questionnaire responses showed exceptionally high values on several points. Based on these recurring patterns, we consider it justified to treat workplace psychological terror as a separate area of research. In our opinion, the results obtained provide a suitable starting point for launching a broader scientific discourse and for more targeted, theoretical, and empirical research into the phenomenon. Our current article can be seen as a pilot study, primarily aimed at exploring whether certain aspects of workplace psychological terror within the sports workplace justify conducting a more in-depth, targeted study in the Hungarian context.

### 1.1 Conceptual approaches to workplace psychological terror

In our research, we focus on the phenomenon of workplace psychological terror within the context of workplace stressors. The term ‘workplace psychological terror’ appears in various translations and interpretations in international and domestic literature, and its use is often influenced by linguistic and cultural context. In this chapter, we examine how the scientific literature addresses the phenomenon in various ways. Kaucsek & Simon (10) refer to Leymann's work, in which he coined the term “mobbing” based on earlier interpretations, in which the term was first used to describe group attacks by animals and then violent group behavior among children. Based on these, he introduced it into the world of the workplace to describe persistent and systematic psychological terror among adults.

Kaucsek & Simon highlight the findings of Leymann’s workplace mobbing, also known as psychological terror, that is a type of conflict in which certain individuals are exposed by their colleagues and/or supervisors – frequently and over a prolonged period – to various forms of mobbing actions (10).

Ballard & Easteal (11) observed that workplace bullying is referred to by a multitude of terms in English-language literature. While mobbing is often highlighted as a significant term, alongside bullying and harassment, this diverse vocabulary underscores the complexity of the phenomenon and the ongoing challenge of arriving at a universally clear and unambiguous definition.

In this present study, the term workplace psychological terror is used as the primary terminological framework. The interpretation of the phenomenon in the context of sports is grounded in the definition used by Kaucsek & Simon [(10), p. 541], Leymann [(12), p. 119], and Juhász [(13), p. 9]. However, in the literature review and citations, we retain and indicate the original terminology used by the representative authors, such as mobbing, workplace bullying, or other related definitions, in order to preserve conceptual accuracy and ensure scientific consistency. Hajdú (14) highlights that the origin of the word bullying stems from Andre Adams and Tim Field, who used the expression workplace bullying instead of Leymann's mobbing in the workplace concept. The author also states that some researchers claim that mobbing and bullying are synonyms. Mobbing is defined by Schwickerath & Holz (15) as a form of workplace communication characterized by conflict, where a vulnerable individual is subjected to harassment, consistently humiliated, and excluded by one or more colleagues over an extended period, and is also subjected to direct or indirect attacks. In the publication by Schwickerath & Holz (15), the authors reference the work of Zapf (16), who expanded on Leymann's research and identified six categories of mobbing strategies. These categories include the inappropriate use of organizational tools, social isolation, personal and private attacks that harm an individual's reputation, verbal threats or physical violence, and the spreading of gossip.

An explicitly sports-oriented study by Bachand (17) says that “[…] bullying can takes the form of physical abuse […], emotional abuse […], social abuse […], the theft or damage of an individual's property that is being victimized, or the use of a form of electronic device to produce the results of abuse on social media to the victim.” According to McMahon et al. (18), psychological abuse can be manifested through various methods. Several sports organizations are actively addressing the issue of abuse affecting athletes, even at the highest levels of international competition. The International Olympic Committee's 2016 guidelines for IFs and NOCs specify how to develop and implement policies to protect athletes from harassment and abuse, and define five categories: harassment, psychological abuse, physical abuse, sexual harassment, and neglect (19).

According to the definition by Blanz et al. [(20), p. 369] in the psychology lexicon, psychological terror is primarily a method used in the political arena. Its purpose is to intimidate opponents and render them incapable of action by psychological means such as intimidation and threats. Our study investigates and demonstrates that psychological terror is also present in sports. Interestingly, the study by Gift and Miner (21) suggests that both sports and politics provide valuable platforms for examining how individual actions, economic structures, and public perception influence the roles and expectations of individuals within these systems. They are both highly visible and influential spheres, drawing implicit parallels to workplace dynamics and professional conduct.

By using the term workplace bullying to describe the phenomenon, Nielsen et al. (22) define it within the framework of organizational and occupational psychology (OOP) as repeated and persistent negative acts occurring in the context of a power imbalance, thereby distinguishing it from a single conflict or isolated incident. Workplace bullying is further characterized by Nielsen et al. (23) as a systematic and sustained form of mistreatment, indicating underlying organizational dynamics. According to Gilioli et al. (24), various forms of psychological terror can be observed in many workplaces. The findings of Juhász (13) show that the elements of workplace psychological terror are closely tied to work-related stress. Juhász's [(13), pp. 7–9] study indicates workplace psychological terror is listed within the group of workplace stressors that are classified as stressors related to the role played within the organization. Within this system created by Juhasz, these stressors primarily appear at the group level and belong to the category of conflicts within the group.

Kaucsek & Simon [(10), pp. 541–543] highlight that through his research on workplace psychological terror, Leymann compiled a list of 45 mobbing actions known as the Leymann Inventory of Psychological Terror (LIPT). According to this framework, psychological terror is considered to exist when someone is confronted with one or more items from this list at least once a week and continuously, over a period of six months. The terminologies used in our study, such as bullying, sexual abuse, psychological abuse, physical abuse, sexual harassment, neglect, harassment, abuse, mockery, humiliation, insult, non-accidental violence, verbal abuse, fear of punishment, coercion to perform prohibited acts, negative discrimination, sexual harassment, and emotional manipulation, as well as additional definitions found in international literature, can all be classified within the framework of the 45 mobbing actions attributed to Leymann, as described by Kaucsek & Simon [(10), p. 543]. These behavioral manifestations are consistent with LIPT and can be understood as psychological terror in sports settings. We are also going to include positive discrimination, although it does not appear among Leymann's 45 mobbing actions. It can nonetheless manifest in sporting contexts, where preferential treatment of one individual may indirectly disadvantage those who do not receive it, while also exposing the favored individual to negative reactions or social exclusion from the broader group.

The athlete experiences identified through the qualitative interviews and subsequently examined in the questionnaire can be conceptually mapped onto the behavioral manifestations described by Kaucsek & Simon [(10), p. 543] as constituting Leymann's mobbing actions. Although the present questionnaire was not designed as a direct adaptation of Leymann's typology, the nine categories developed for the study were derived from athletes’ reported experiences and the sport-specific manifestations of workplace psychological terror and were organized into conceptually coherent groups. Accordingly, the categorization remains consistent with the theoretical framework of workplace psychological terror while reflecting the characteristics of the sporting environment.

Our research examines sport as an atypical workplace, meaning forms of employment that differ from standard employment relationships, which are usually full-time, permanent contracts characterized by a relationship of subordination and occupational safety. Atypical employment, on the other hand, includes forms that lack one or more fundamental characteristics. These include temporary, part-time, or non-standard contracts, which differ from forms of employment traditionally considered stable (25). Using this approach, we interpret and analyze the phenomenon of workplace psychological terror as a problem in sports, occurring in an atypical workplace environment. This approach allows us to interpret the phenomenon within the specific organizational and hierarchical structure of sport.

### 1.2 The role of human resources (HR) in organizations

From our perspective, sport represents an unconventional form of employment. Therefore, we explored the issue of workplace psychological terror from a fresh angle, specifically through the lens of Occupational and Organizational Psychology (OOP). This approach enables a wider interpretation of systematic behavioral patterns. In this study, we define the workplace in terms of organizational structure rather than legal context. Regardless of their contractual employment status, athletes operate within structured organizations. These organizations are characterized by formal leadership, established rules, hierarchical decision-making processes, performance evaluations, and dependent relationships with coaches and sports organizations. These features closely align with the organizational environments examined in OOP, making this framework suitable for investigating workplace psychological terror in the realm of sports.

It has been proven in many areas that human resource management is of paramount importance in the life of an organization [(26), p. 16]. This also applies to sports as an atypical workplace. The findings of Juhász (13) suggest that psychological terror within the workplace can significantly harm the physical and mental health of employees, thereby adversely affecting the organization's performance and efficiency. As introduced in the findings of Resperger et al. (9), sport is a workplace environment where psychological stress can cause lasting damage to athletes’ long-term mental health. Furthermore, it adversely affects the performance of the sport and negatively impacts the sporting community. This is in line with the findings of Nielsen et al. (23), who indicate that the occurrence of bullying can show a poor and detrimental psychosocial working environment, as it is a complex psychosocial and organizational issue. Bullying goes beyond individual conflicts because it highlights there are issues with the organizational environment, support systems, and leadership in shaping both experiences and the consequences for employees.

The economic events of the last few years have made company managers and human resources professionals realize that HR management is as important (if not more important) in the life of an organization as the production of profit (26). A key part of HR management is the organizational function that involves developing and implementing human resource strategies, as well as preventing and resolving people-related issues. Three essential features of this resource need to be highlighted. Firstly, human resources need to be managed in the same way as any other resource. Secondly, human resource management has become strategic and, thirdly, it is not only a service provider and coordinator, but also an advisor (27). It harmonizes and aligns the organization's aspirations and the expectations of its employees (28).

According to Salin (29), several key HR measures, such as written policies and designated responsibilities, can be adopted to prevent bullying. Research by Bowen & Ostroff (30) shows that well-designed human resources management (HRM) frameworks create positive psychological environments that boost engagement and performance. This highlights the reciprocal relationship between HR practices and the principles of OOP. Hajdú [(14), pp. 79–81] also highlights that mobbing typically occurs in work environments where working methods are poorly coordinated and where leadership is inattentive or ineffective. Victims usually have high-level competencies, such as dedication and integrity. His finding also indicates that in Hungarian legislation, mobbing is not specifically regulated.

### 1.3 Aim of the study

The purpose of this research was to examine the prevalence and patterns of workplace psychological terror in the sporting environment among Hungarian athletes. The study focused on several mobbing actions of workplace psychological terror, including treatment violating human dignity, verbal and physical abuse, fear of punishment, coercion, discrimination, sexual harassment, and emotional manipulation. Our first aim was to identify the groups most susceptible to such experiences by analyzing differences across gender, age groups, sport types, and competition levels. In addition, we sought to determine the main sources of workplace psychological terror and to explore their socio-demographic variations. The research aimed to highlight that if we consider the sports environment to be an atypical workplace, then several corporate solutions can be applied to reduce cases of psychological harassment and improve workplace culture.

Our additional objective was to examine whether, based on our results, further development and validation of the measurement tool are justified, considering the specific characteristics of the Hungarian sports workplace environment. Our study did not aim to settle the topic but to add to a scientific discussion that could lay the groundwork for more in-depth, methodologically solid domestic research on workplace harassment in the future.

## 2 Materials and methods

### 2.1 Participants and procedure

The research was conducted between October 2024 and April 2025 using an online questionnaire administered via the SurveyMonkey platform. The survey was widely distributed among athletes, sports organizations, and university students. Completion required approximately 15–20 minutes, and responses were collected anonymously. Before participation, all respondents were informed about the purpose of the study, assured that their involvement was voluntary, and notified that no personal data would be collected. The study was carried out in accordance with the principles of the Declaration of Helsinki and followed the guidelines of the Ethics Committee of the Hungarian University of Sports Science (Ethical number: MTSE-OKE-KEB/03/2023).

Overall, 319 responses were collected. Of these, 225 were suitable for our analysis. 94 responses were excluded due to incomplete information, resulting in a small reduction in the final sample size. Among the participants, 127 were male (56%), and 98 were female (44%). We asked the participants about their age category, and most of the participants were between 18 and 29 (*n* = 149; 66%). Furthermore, 15 (6%) participants were aged between 30-39; 17 (8%) of them were aged between 40-49; 15 (6%) of them were aged between 50-59, and 29 (14%) of the participants were aged above 60 + . Several sports were represented in this sample. However, due to fragmentation, we divided them into individual (*n* = 128; 57%) and team sports (*n* = 96; 43%). The competition level of the participants was categorized as the following: Elite (68; 30.2%), representing athletes competing at the highest professional or Olympic level; International (42; 18.7%), including those who regularly participate in international tournaments and events; Domestic (88; 39.1%), referring to athletes active primarily in national leagues and competitions; and Youth (27; 12.0%), encompassing junior athletes competing in age-group categories. Although not all athletes included in the present study were employees in the legal sense, all participants operated within organized sport systems characterized by formal authority relationships, performance expectations, organizational hierarchies, and structured supervision. In the present study, the workplace framework is therefore used as a functional organizational concept rather than a strictly legal employment category. Youth athletes generally participate within structured sports organizations under formal coaching supervision and institutional authority. Although they may not have contractual employment relationships, they are exposed to many of the same organizational hierarchies, power asymmetries, and performance expectations as adult athletes. Therefore, they were included because the study focuses on organizational experiences rather than legal employment.

### 2.2 Measures

The questionnaire was developed specifically for the present study following a multi-stage process that combined evidence from the literature with the practical experiences of athletes. The development of the questionnaire followed a predominantly inductive approach informed by existing theoretical frameworks. First, a review of the relevant literature on workplace psychological terror, workplace bullying, athlete abuse, and safeguarding in sport was conducted to identify the principal forms of workplace psychological terror reported in previous research. These findings served as the initial conceptual framework for our questionnaire development. In the second stage, the preliminary questionnaire was received through approximately one-hour semi-structured interviews with 10 athletes representing a broad range of sporting backgrounds. The interviewees encompassed competitive athletes, Olympians, former competitors, and esteemed coaches. When selecting interviewees, the research team deliberately aimed to achieve a balanced gender representation and to include representatives of both team sports and individual sports within the sample. The interviews were used to better understand workplace psychological terror.

During these interviews, participants evaluated the clarity, relevance, and completeness of the questionnaire items, shared additional personal experiences, and identified ambiguous or missing content. Based on their feedback, several items were revised, clarified, supplemented, or removed before the questionnaire was finalized. Using these methods, we identified nine dimensions of athlete maltreatment: treatment violating human dignity, verbal abuse, fear of punishment, negative discrimination, positive discrimination, sexual harassment, physical abuse, coercion into prohibited actions, and emotional manipulation.

Following the qualitative refinement, the revised questionnaire underwent a pilot test involving 10 athletes, including four of the previously interviewed participants and six additional participants. The pilot focused on item comprehensibility, questionnaire flow, completion time, and technical functionality. Based on the pilot feedback, the remaining ambiguities and technical issues were corrected before the main data collection commenced. This iterative development process combined literature-based theoretical foundations with athlete-informed practical experience to maximize the content validity and contextual relevance of the instrument within the Hungarian sporting environment.

The first part of the questionnaire included demographic questions. Respondents were asked to provide information about their gender, age, and current place of residence using a closed, multiple-choice format. This was followed by a question about their sport and current practices, in which they could select from a predefined list or provide a free-text response if their sport was not listed. The next questions focused on sport achievements and their current relationship with sport.

The questionnaire included various questions about forms of abuse, which were organized into nine categories that addressed different types of mobbing actions within the context of workplace psychological terror. Each category of mobbing action contained three questions designed to assess the athlete's perception of that specific mobbing action. The response options were as follows: 1. Yes, I have experienced it; 2. Yes, I have experienced it, and I know of other cases; 3. I have not experienced it, but I know of someone who has; 4. I have not experienced it at all. In the second part of the questionnaire, participants who reported experiencing abuse were asked to specify the exact forms of harassment they encountered, such as mocking, humiliation, or insults, and they could select multiple responses. They were also asked to identify the sources of psychological terror, including coaches, teammates, or parents, with the option to select multiple answers. Additionally, an open-ended “Other” option was provided in each section, allowing respondents to give written input if they desired.

This design enabled a detailed exploration of both the nature and context of workplace psychological terror in sport. The results of each category are presented in Supplementary Tables S1–S9. The present instrument was not developed as a traditional psychometric scale measuring a single latent construct. Instead, it was designed as a multidimensional prevalence assessment tool capturing distinct categories of workplace psychological terror experiences. Therefore, classical scale validation procedures such as confirmatory factor analysis were not considered primary validation strategies. Content validity was established through an iterative development process consisting of literature review, qualitative interviews with experienced athletes, expert-informed refinement, and pilot testing prior to the main data collection, with all items grounded in established theoretical frameworks (31).

For statistical analysis, the original four response categories were recoded into two broader groups: “experienced” and “not experienced.” The “experienced” category included respondents who reported personal experience, either alone or in combination with awareness of other cases, as well as respondents who reported indirect awareness through a direct victim. The “not experienced” category included only respondents who reported no experience or awareness of the given category. This dichotomization was undertaken to facilitate exploratory categorical group comparisons using chi-square tests. Because retaining all four response categories would have resulted in small cell frequencies in several subgroup analyses, especially across age groups and competition levels, dichotomization was used to provide more stable and interpretable group comparisons. Given the study's primary objective of identifying exposure patterns rather than differentiating degrees of indirect awareness, collapsing the categories ensured adequate statistical power, minimized sparse cell frequencies, and improved model stability. The intraclass correlation coefficient (ICC = 0.53) reflected moderate consistency across the nine dimensions. As the instrument was conceptualized as a multidimensional prevalence assessment tool rather than a unidimensional psychometric scale, a high level of internal consistency was not anticipated.

### 2.3 Statistical analysis

Descriptive statistics (means, standard deviations, frequencies, and percentages) were calculated to provide an overview of the sample characteristics and the distribution of responses across nine categories of workplace psychological terror. For the prevalence analysis, the original four response categories were retained to distinguish between personal experience, personal experience combined with awareness of other cases, indirect awareness through a direct victim, and no reported experience. For the group comparison analyses, the response categories were dichotomized into two broader groups: “experienced” and “not experienced.” The “experienced” category included respondents who reported personal experience, personal experience combined with awareness of other cases, or indirect awareness through a direct victim. The “not experienced” category included only respondents who reported no experience or awareness of the given category. This dichotomization was applied because retaining all four response categories would have resulted in small cell frequencies in several subgroup analyses, particularly across age groups and competition levels. Therefore, the dichotomized analyses were used to provide more stable and interpretable exploratory group comparisons. Group differences were examined using chi-square tests (*χ*^2^) of independence to compare the nine workplace psychological terror categories across socio-demographic and sport-related variables, including gender, age group, sport type, and competition level. In addition, one-way analyses of variance (ANOVA) with Tukey's *post-hoc* tests were used to examine differences in the reported sources of workplace psychological terror across athlete characteristics. All analyses were conducted using Jamovi 2.3, an open-source statistical software package. Statistical significance was set at *p* < .05.

## 3 Results

### 3.1 Prevalence of workplace psychological terror categories

Analysis of workplace psychological terror-related mobbing actions (additionally adding positive discrimination as an occurring factor in sports) was organized into nine categories, showing that Hungarian athletes reported a broad range of harmful experiences during their athletic careers (Table 1; see also Supplementary Tables S1–S9). Based on personal exposure, the most frequently reported category was verbal abuse (66.0%), followed by positive discrimination (56.3%), fear of punishment (55.1%), and treatment violating human dignity (53.0%). Negative discrimination was also reported by nearly half of the respondents (47.1%), while emotional manipulation was reported by more than one-third of athletes (35.3%).

**Table 1:** Prevalence of the nine categories of workplace psychological terror among Hungarian athletes.

Category	Personally experienced	Personally experienced and knew other cases	Indirect awareness only	Not experienced
Treatment violating human dignity	45 (20.0%)	74 (33.0%)	57 (25.0%)	49 (22.0%)
Verbal abuse	63 (28.0%)	86 (38.0%)	26 (12.0%)	50 (22.0%)
Fear of punishment	66 (29.3%)	58 (25.8%)	19 (8.4%)	82 (36.4%)
Negative discrimination	49 (22.0%)	56 (25.1%)	22 (9.9%)	96 (43.1%)
Positive discrimination	71 (32.0%)	54 (24.3%)	30 (13.5%)	67 (30.2%)
Sexual harassment	12 (5.4%)	11 (5.0%)	18 (8.1%)	180 (81.5%)
Physical abuse	18 (8.1%)	28 (12.7%)	26 (11.8%)	149 (67.4%)
Coercion into prohibited activities	3 (1.4%)	9 (4.1%)	14 (6.3%)	195 (88.2%)
Emotional manipulation	37 (16.7%)	41 (18.6%)	14 (6.3%)	129 (58.4%)

The supplementary results provide further detail on the specific forms and sources of these experiences. Treatment violating human dignity most often involved mocking, humiliation, exclusion, or rumor-spreading, with teammates/sporting partners and coaches most frequently identified as sources. Fear of punishment commonly included being sent home from training, exclusion from the team, or intentionally excessive training as punishment, and was most often linked to coaches. Discrimination-related experiences were frequently associated with reputation, athletic achievements, or physical attributes, with coaches most often identified as responsible.

Sexual harassment (10.4%), physical abuse (20.8%), and coercion into prohibited activities (5.5%) were reported less frequently than the other categories, but they were still present in the sample. These findings indicate that more severe or legally sensitive forms of workplace psychological terror were not absent from the Hungarian sport context, even if their prevalence was lower. Overall, the results suggest that workplace psychological terror appears across multiple forms in sport, with coaches and teammates/sporting partners emerging as central sources in several categories.

### 3.2 Group differences by athlete characteristics

For the group comparisons presented in Table 2, the original four response categories were dichotomized into “Experienced” and “not Experienced” categories. This decision was made because retaining all four response options would have resulted in small cell frequencies in several subgroup analyses, particularly across age groups and competition levels. Therefore, the chi-square analyses should be interpreted as exploratory group comparisons indicating whether the reporting of each workplace psychological terror category differed across athlete characteristics.

**Table 2:** Descriptives of categorized workplace mobbing actions within sports workplace psychological terror by gender, age, sport type, and competition level.

Independent variable		Treatment violating human dignity			Verbal abuse			Fear of punishment			Negative discrimination			Positive discrimination			Sexual harassment			Physical abuse			Coercion into prohibited actions			Emotional manipulation		
	Category (N)	Not experienced (*n* = 49)	Experienced (*n* = 176)	*χ* ^2^	Not experienced (*n* = 50)	Experienced (*n* = 175)	χ^2^	Not experienced (*n* = 82)	Experienced (*n* = 143)	χ^2^	Not experienced (*n* = 96)	Experienced (*n* = 127)	χ^2^	Not experienced (*n* = 67)	Experienced (*n* = 155)	χ^2^	Not experienced (*n* = 180)	Experienced (*n* = 41)	χ^2^	Not experienced (*n* = 149)	Experienced (*n* = 72)	χ^2^	Not experienced (*n* = 195)	Experienced (*n* = 26)	χ^2^	Not experienced (*n* = 129)	Experienced (*n* = 92)	χ^2^
Gender	Men (127)	29 (23%)	98 (77%)	0.19	29 (44%)	98 (56%)	0.06	48 (59%)	79 (55%)	0.23	59 (61%)	66 (52%)	2.00	37 (55%)	87 (56%)	0.02	104 (58%)	19 (46%)	1.77	85 (57%)	38 (47%)	0.36	105 (54%)	18 (69%)	2.20	78 (60%)	45 (49%)	2.90
	Women (98)	20 (20%)	78 (80%)		21 (42%)	77 (44%)		34 (41%)	64 (45%)		37 (39%)	61 (48%)		30 (45%)	68 (44%)		76 (42%)	22 (54%)		64 (73%)	34 (43%)		90 (46%)	8 (31%)		51 (41%)	47 (51%)	
Age	18–29 (149)	21 (14%)	128 (86%)	**30**.11*	29 (58%)	120 (69%)	6.86	58 (71%)	91 (64%)	4.91	61 (64%)	87 (69%)	7.68	33 (49%)	115 (74%)	**37**.43***	119 (66%)	29 (71%)	8.14	113 (76%)	35 (49%)	**18**.61***	128 (66%)	20 (77%)	7.77	87 (67%)	61 (66%)	2.90
	30–39 (15)	2 (13%)	13 (87%)		1 (2%)	14 (8%)		2 (2%)	13 (9%)		4 (4%)	11 (9%)		2 (3%)	13 (8%)		12 (7%)	3 (7%)		5 (3%)	10 (14%)		12 (6%)	3 (12%)		6 (5%)	9 (10%)	
	40–49 (17)	4 (24%)	13 (76%)		5 (10%)	12 (7%)		6 (7%)	11 (8%)		5 (5%)	11 (9%)		3 (4%)	13 (8%)		10 (6%)	6 (15%)		8 (5%)	8 (11%)		13 (7%)	3 (12%)		9 (7%)	7 (8%)	
	50–59 (15)	5 (33%)	10 (67%)		5 (10%)	10 (6%)		7 (9%)	8 (6%)		8 (8%)	7 (6%)		12 (18%)	3 (2%)		13 (7%)	2 (5%)		8 (5%)	7 (10%)		15 (8%)	0 (0%)		9 (7%)	6 (7%)	
	60+ (29)	17 (59%)	12 (41%)		10 (20%)	19 (11%)		9 (11%)	20 (14%)		18 (19%)	11 (9%)		17 (25%)	11 (7%)		26 (14%)	1 (2%)		15 (10%)	12 (17%)		27 (14%)	0 (0%)		18 (14%)	9 (10%)	
Individual/Team	Individual (128)	38 (30%)	90 (70%)	**10**.67*	34 (68%)	94 (54%)	3.10	51 (62%)	77 (54%)	1.35	67 (70%)	60 (48%)	**10**.94***	52 (78%)	74 (48%)	**16**.65***	102 (57%)	23 (57%)	0.01	77 (52%)	48 (67%)	**4**.23*	114 (59%)	11 (42%)	2.53	72 (56%)	53 (58%)	0.04
	Team (96)	11 (12%)	85 (88%)		16 (32%)	80 (46%)		31 (38%)	65 (46%)		29 (30%)	66 (52%)		15 (22%)	80 (52%)		78 (43%)	17 (43%)		71 (48%)	24 (33%)		80 (41%)	15 (58%)		56 (44%)	39 (42%)	
Competition level	Elite (68)	22 (32%)	46 (68%)	**8**.34*	16 (32%)	52 (30%)	2.96	18 (22%)	50 (35%)	4.41	29 (31%)	39 (30%)	1.46	29 (43%)	39 (25%)	**9**.93*	56 (31%)	12 (29%)	3.44	32 (21%)	36 (50%)	**27**.53***	62 (32%)	6 (23%)	3.33	34 (26%)	34 (47%)	5.45
	International (42)	8 (19%)	34 (81%)		13 (26%)	29 (17%)		17 (21%)	25 (17%)		21 (22%)	20 (16%)		6 (9%)	35 (23%)		30 (17%)	10 (24%)		24 (16%)	16 (22%)		32 (16%)	8 (31%)		21 (26%)	19 (21%)	
	Domestic (88)	12 (14%)	76 (86%)		16 (16%)	72 41%)		37 (45%)	51 (36%)		35 (36%)	52 (41%)		24 (36%)	62 (40%)		69 (38%)	17 (41%)		74 (50%)	12 (17%)		77 (39%)	9 (35%)		58 (45%)	28 (30%)	
	Youth (27)	7 (26%)	20 (74%)		5 (19%)	22 (13%)		10 (12%)	17 (12%)		11 (11%)	16 (13%)		8 (12%)	19 (12%)		25 (14%)	2 (5%)		19 (13%)	8 (11%)		24 (12%)	3 (12%)		16 (12%)	11 (12%)	

Bold values indicate statistically significant results.

*p < .05, **p < .01, ***p < .001.

Overall, the clearest group differences were observed by age, sport type, and competition level, whereas gender differences were less consistent and did not reach statistical significance across the nine categories. Age was significantly associated with treatment violating human dignity (*p* < .001), positive discrimination (*p* < .001), and physical abuse (*p* < .001). Descriptively, younger athletes, particularly those in the 18–29 and 30–39 age groups, reported higher levels of several experiences than older athletes. Sport type was significantly associated with treatment violating human dignity (*p* = .001), negative discrimination (*p* < .001), positive discrimination (*p* < .001), and physical abuse (*p* = .040), indicating that the pattern of reported experiences differed between individual- and team-sport athletes. Competition level was also significantly associated with treatment violating human dignity (*p* = .039), positive discrimination (*p* = .019), and physical abuse (*p* < .001), suggesting that exposure patterns varied across elite, international, domestic, and youth sport contexts. Overall, these findings indicate that reported experiences of workplace psychological terror varied more clearly by age, sport context, and competition level than by gender*.*

### 3.3 Reported sources of workplace psychological terror

Table 3 presents the one-way ANOVA results for reported sources of workplace psychological terror by gender, age group, sport type, and competition level. The results showed that the reported sources of workplace psychological terror differed across several athlete characteristics.

**Table 3:** One-way ANOVA results for workplace psychological terror by source and athlete characteristics.

Independent variable	Group	Coach	Teammates	Parent	Sports leader	Private relationship	Media	Competition staff	Other team member	Other
Gender	Men (*N* = 126)	3.15 (2.39)	2.37 (2.02)	0.71 (1.47)	0.90 (1.59)	0.49 (1.22)	0.17 (0.78)	0.33 (0.90)	0.25 (0.83)	0.16 (0.72)
	Women (*N* = 97)	3.87 (2.34)	1.53 (1.53)	0.63 (1.18)	0.96 (1.72)	0.29 (0.82)	0.34 (1.03)	0.24 (0.63)	0.15 (0.42)	0.13 (0.53)
	F	5.03*	12.44***	0.19	0.06	2.20	1.74	0.74	1.36	0.09
Age	18–29 (*N* = 148)	3.48 (2.30)	2.30 (1.85)	0.69 (1.33)	0.66 (1.42)	0.47 (1.15)	0.17 (0.71)	0.26 (0.83)	0.22 (0.79)	0.20 (0.76)
	30–39 (*N* = 15)	4.73 (2.60)	3.33 (2.32)	1.27 (2.37)	2.33 (2.23)	1.00 (1.77)	0.87 (1.81)	0.60 (0.74)	0.53 (0.52)	0.07 (0.26)
	40–49 (*N* = 17)	3.94 (2.73)	1.41 (1.54)	1.06 (1.39)	1.53 (2.35)	0.12 (0.33)	0.71 (1.61)	0.53 (1.01)	0.35 (0.49)	0.00 (0.00)
	50–59 (*N* = 15)	2.80 (2.27)	1.00 (1.13)	0.60 (0.91)	1.60 (2.03)	0.07 (0.26)	0.33 (0.62)	0.13 (0.35)	0.07 (0.26)	0.13 (0.52)
	60+ (*N* = 28)	2.75 (2.38)	0.61 (0.92)	0.07 (0.26)	0.89 (1.13)	0.07 (0.26)	0.00 (0.00)	0.21 (0.57)	0.00 (0.00)	0.00 (0.00)
	F	1.87	15.78***	9.27***	3.04*	4.60**	—	1.54	—	—
Individual/ team	Individual (*N* = 128)	3.30 (2.51)	1.55 (1.72)	0.74 (1.40)	1.06 (1.86)	0.34 (1.14)	0.28 (1.02)	0.35 (0.94)	0.16 (0.77)	0.13 (0.68)
	Team (*N* = 94)	3.68 (2.22)	2.57 (1.88)	0.57 (1.28)	0.76 (1.28)	0.49 (0.97)	0.20 (0.71)	0.20 (0.52)	0.28 (0.54)	0.18 (0.60)
	F	1.39	17.48***	0.86	2.12	1.17	0.46	2.27	1.64	0.42
Sport level	Elite (*N* = 67)	3.90 (2.43)	1.37 (1.64)	0.73 (1.33)	1.51 (1.92)	0.33 (0.94)	0.42 (1.17)	0.39 (0.76)	0.24 (0.50)	0.09 (0.34)
	International (*N* = 42)	3.21 (2.68)	2.19 (2.09)	0.90 (1.71)	1.00 (2.12)	0.64 (1.72)	0.33 (1.16)	0.52 (1.33)	0.33 (1.26)	0.26 (1.11)
	Domestic (*N* = 87)	3.18 (2.11)	2.23 (1.80)	0.54 (1.27)	0.48 (0.90)	0.33 (0.80)	0.14 (0.57)	0.11 (0.36)	0.16 (0.45)	0.17 (0.61)
	Youth (*N* = 27)	3.67 (2.62)	2.52 (1.95)	0.59 (0.93)	0.81 (1.55)	0.44 (0.75)	0.04 (0.19)	0.22 (0.70)	0.11 (0.32)	0.04 (0.19)
	F	1.36	4.34**	0.63	5.83***	0.53	3.21*	3.52*	0.96	1.48

Bold values indicate statistically significant results.

*p < .05, **p < .01, ***p < .001.

Regarding gender, significant differences were found for coaches and teammates. Women reported higher scores for coaches as a source of workplace psychological terror than men, F = 5.03, *p* < .05. In contrast, men reported higher scores for teammates than women, F = 12.44, *p* < .001. This suggests that coach-related experiences were more frequently reported by women, whereas peer-related experiences were more frequently reported by men.

Age-related differences were also observed. The clearest difference appeared for teammates, F = 15.78, *p* < .001, with younger athletes reporting higher scores than older athletes. Significant age differences were also found for parents, F = 9.27, *p* < .001, sport leaders, F = 3.04, *p* < .05, and private relationships, F = 4.60, *p* < .01. Descriptively, athletes aged 30–39 years showed the highest scores for several of these sources, although these findings should be interpreted cautiously because some age groups were small.

Sport type was associated only with teammate-related experiences. Team-sport athletes reported higher scores for teammates than individual-sport athletes, F = 17.48, *p* < .001. No significant differences were found between individual- and team-sport athletes for the other source categories.

Competition level was significantly associated with teammates, F = 4.34, *p* < .01, sport leaders, F = 5.83, *p* < .001, media, F = 3.21, *p* < .05, and competition staff, F = 3.52, *p* < .05. Descriptively, youth athletes reported the highest teammate-related scores, whereas elite athletes showed higher scores for sport leaders, media, and competition staff. Overall, the findings indicate that coaches and teammates were central reported sources of workplace psychological terror, but the relative importance of different sources varied according to gender, age, sport type, and competition level.

## 4 Discussion

This study examined the prevalence and patterns of workplace psychological terror in the sporting environment among Hungarian athletes. The findings reveal a high proportion of respondents reporting experiences of workplace psychological terror across all nine categories. A considerable number of athletes indicated exposure to treatment that violates human dignity and verbal abuse. Moreover, coaches and teammates/sporting partners emerged as the most frequently reported sources across several categories of workplace psychological terror. Based on the results, presenting a comprehensive overview of the findings is a crucial step in developing potential solutions. Previous research examining Hungarian athletes has demonstrated that, concerning workplace stressors affecting athletes, individual athletes encounter a greater number of stressors than their team counterparts (9). Consistent with this pattern, the current study's survey results indicate that individual athletes encountered psychological terror, such as verbal abuse and discrimination more frequently than team athletes.

According to Richardson et al. (32), coaches may utilize access to practice and playing time as strategic tools to ensure the team's expectations are met. Such practices may include disciplinary measures such as reducing playing time or temporarily excluding athletes from training sessions. This form of control is particularly relevant in sport, as athletes are typically motivated to train, improve, and compete. In our study, fear of punishment was commonly reported by participants, since most of the participants reported that they had either personally experienced fear of punishment or knew of such cases directly from victims. The most frequently reported forms included being sent home from training, being excluded from the team, and being subjected to intentionally excessive training as a form of punishment (see Supplementary Tables S1–S9). Coaches were most frequently identified as the source of punishment-related fear. Although fear can serve as an extrinsic motivator and may produce short-term performance improvements, its use is linked to harmful long-term effects on both the individual athlete and the culture of the sporting group or community. Research on youth hockey players shows that techniques like punitive coaching behaviors hinder the development of a positive self-image in young athletes and also impact their ability to build constructive relationships with others (33). Therefore, our findings suggest that fear of punishment should be considered an important area for further safeguarding research and coach education.

The public typically associates athletes with aesthetic, muscular physiques. In numerous sports, physical attributes and condition significantly influence performance. Consequently, physical appearance and characteristics (e.g., muscle mass, body weight, height, and other physical features) are central to an athlete's career. Participants who faced negative discrimination indicated that it was primarily based on their physical attributes. Age was identified as the second most common reason for discrimination, followed by concerns related to reputation and athletic achievements. Additionally, women reported experiencing higher levels of discrimination from coaches compared to men in the sample. While this suggests a potential gender difference, it should be interpreted cautiously due to the exploratory nature of the study. The study also examined positive discrimination. More than 60% of respondents reported direct or indirect exposure, most frequently related to reputation and athletic achievements (27.8%). These patterns may suggest that achievement-based differential treatment influences athletes’ experience within the sporting environment. Previous research has demonstrated that discrimination can manifest in various forms across different sports settings (34). However, since organizational culture was not directly assessed, these findings should not be taken as conclusive evidence of broader organizational or cultural characteristics.

Emotional manipulation involving control over the victim is also a common form of workplace psychological terror in sports. Nearly half of the respondents indicated that coaches are the primary perpetrators of emotional manipulation, placing them at the top of the list of abusers. A coach-athlete relationship requires an asymmetrical power dynamic and a tremendous amount of trust from the athlete. In this relationship, even adults find it difficult to recognize when the relationship crosses from professional support to control based on emotional manipulation. It should be noted that levels of emotional manipulation were higher among domestic athletes, suggesting that this area may warrant further safeguarding research and the development of preventive coaching practices within the Hungarian sports context. On a more favorable note, age-related analyses revealed that athletes in the 30–39 age group reported significantly lower levels of emotional manipulation compared to older cohorts, suggesting lower reported exposure or differences in reporting patterns at this stage of athletes’ careers. However, the present study cannot determine the underlying reasons for the observation.

The total number of cases involving direct victims or individuals with firsthand knowledge indicates that the lowest incidences were reported for sexual harassment, physical abuse, and coercion to use prohibited substances. Simultaneously, it is important to recognize that, despite higher numbers observed for other categories, these are nonetheless significant and further corroborate the findings regarding the seriousness of the problem. Interestingly, athletes in the 40–49 age group reported sexual harassment at a higher rate than other age groups. Although this study cannot determine the reasons for this pattern, one possible explanation suggested in previous literature is that older athletes may have been socialized in periods when awareness and prevention policies were less developed, leaving them more exposed to abusive practices during their careers (1, 4). In addition, generational differences in disclosure may also play a role, as individuals in this age group might be more willing to retrospectively acknowledge and report experiences that were normalized or silenced at the time (35).

Regarding the reported sources of workplace psychological terror, respondents were asked to identify the individuals or groups involved in each category. Across the nine categories, coaches, teammates or sporting partners, and sport leaders were the most frequently reported sources. Coaches were identified as the most frequent source in eight of the nine categories, while teammates or sporting partners were also repeatedly reported, particularly in relation to treatment violating human dignity. These findings suggest that workplace psychological terror in sport should be examined not only as an individual coach–athlete issue, but also in relation to peer interactions and organizational roles. However, because organizational culture, leadership practices, and safeguarding mechanisms were not directly measured, the findings should be interpreted as reported source patterns rather than evidence of organizational or cultural causality. Previous organizational psychology and sport management research similarly suggests that workplace bullying and non-accidental violence may be shaped by broader organizational environments and leadership practices (23, 36). Teammates and sports partners were ranked among the top 3 abusers in all questions.

On the dimension of treatment violating human dignity, based on the questionnaire items referring to being picked on, mocked, humiliated, ostracized, teased, or having rumors spread about them, respondents most frequently identified teammates as the harassers (40,9% of the responders). These findings suggest that peer interactions may represent an important context in which athletes report experiences of workplace psychological terror. While organizational or team culture was not directly assessed, previous literature indicates that repeated interpersonal maltreatment may reflect broader cultural or organizational processes (23). The results revealed that this type of treatment, violating human dignity, was one of the most frequently reported forms of workplace psychological terror among Hungarian athletes. Furthermore, it turned out that the younger athletes were significantly more vulnerable to it. Regarding age, athletes within the age brackets of 18–29 years and 30–39 years also reported experiencing a higher incidence of treatment violating their human dignity compared to older age cohorts. Further studies should investigate the organizational and contextual factors that may contribute to these observed patterns.

Sports leaders were ranked among the top four in each mobbing action category, and they were identified as perpetrators of forms of workplace psychological terror. This finding is especially concerning, as sports leaders are responsible for shaping the organizational culture, setting a genuine example, and ensuring a psychologically healthy sporting environment. According to Sinek (37), numerous threats are exemplified by factors that lie outside an organization's control and are in continual flux, such as the unpredictability inherent in the economic environment or the emergence of new competitors within the marketplace. Conversely, organizations possess considerable influence over internal conditions, among which the quality of leadership and the execution of managerial responsibilities are paramount. When leaders prioritize the safety and well-being of their employees, the organizational climate benefits from an enhanced sense of belonging and psychological security. Applying Sinek's (37) leadership framework to sport, where leaders are expected to promote belonging and psychological safety, it is noteworthy that sports leaders were identified by some respondents as reported sources of workplace psychological terror. This observation warrants further investigation in future research.

Interestingly, athletes competing at the elite and junior levels reported significantly higher levels of discrimination than those participating at the national level. Sources of discrimination included not only teammates but also sports managers, the media, and competition organizers, indicating that maltreatment at these levels may involve multiple actors operating across different levels of the sporting environment. The findings align with previous studies demonstrating that athletes at higher levels of competition are particularly vulnerable to abuse due to intense performance demands, hierarchical power structures, and increased media scrutiny (35). Athletes and those competing at the domestic level appear most at risk, emphasizing the urgent need for targeted safeguarding interventions in both grassroots and youth sport in Hungary. From an occupational and Organizational Psychology perspective, these findings support considering organizational-level safeguarding strategies alongside individual interventions. However, because organizational culture and governance were not directly assessed, the present study cannot determine whether these factors explain the reported experiences.

Our study's findings show that many athletes have reported experiences of workplace psychological terror. These findings suggest that current safeguarding and organizational practices may not always be perceived as sufficient to prevent such experiences. However, the present study did not directly evaluate organizational culture, HR practices, or governance structures. Therefore, conclusions regarding the organizational origins of these reported experiences should be interpreted with caution. Nevertheless, our findings are consistent with previous organizational psychology research that repeated workplace bullying and psychological abuse may be influenced by broader organizational environments and leadership practices (23, 36). Future research should directly examine these organizational factors to determine their contribution to workplace psychological terror in sport. Certain HRM practices have been proposed in previous literature as potentially contributing to the prevention of workplace psychological terror. The critical framework of HR Management can assist in understanding and implementing measures of prevention by concentrating on specific policies, HR practices, and the role of HR professionals within an organization (29). This approach may also apply to various organizations operating within the sporting environment. The findings of Yang & Mostafa (38) show that organizational culture can shape perceptions of HR practices and influence how HR functions within the sector. Leaders and managers are encouraged to foster a positive culture to enhance the benefits of HR practices.

We believe our study provides useful data about workplace psychological terror among Hungarian athletes, however, there are still some limitations that should be addressed. First, the data were collected using a self-administered online questionnaire, which may introduce self-report bias and issues related to recall accuracy. Respondents may have underreported or overreported their experiences of harassment due to stigma, social desirability, or differences in personal interpretation of the items. Furthermore, several other variables should be included in future studies. For example, we did not ask about forms of online harassment from the respondents, which also diminishes the generalizability of this study. We consider our research to be a preliminary study that could lead to future studies to address these gaps and improve understanding of athletes facing workplace psychological terror within the sport context. An additional limitation concerns the subgroup analyses. Relatively small and unequal sample sizes represented several age categories and competition-level groups. Consequently, these subgroup comparisons should be interpreted with caution as the limited sample sizes may have reduced statistical power and increased uncertainty of observed differences. Therefore, the subgroup findings should be regarded as exploratory and require confirmation in larger, more balanced samples. The workplace framework adopted in this study should not be interpreted as implying that all athlete groups share identical employment conditions. Rather, it provides an organizational perspective for examining hierarchical relationships, power asymmetries, workplace stressors, and safeguarding challenges across different sporting contexts. Future studies may benefit from comparing athletes with different contractual or employment statuses more explicitly.

## 6 Conclusion

It is imperative to address this topic using an interdisciplinary approach, which is recommended to safeguard the mental and physical well-being of athletes and to foster safe and supportive interactions within the sporting community. Several Hungarian sports organizations are dedicated to ensuring the physical and mental safety of athletes by formulating various guidelines for dealing with harassment and abuse (39). Continuous education and the provision of information on various platforms for individuals working in sports and those wishing to participate in sports are essential for prevention. Sports federations are striving to fulfill their obligation to inform the sporting community, and various sports organizations are also contributing to raising awareness through the dissemination of educational materials. However, the question remains as to whether they can implement appropriate measures concerning this issue, based on the outcomes.

An effective HR strategy involves adopting a multilateral approach that encompasses sport as a workplace, recognizing it as an environment where individuals operate. The present findings suggest that systemic approaches may contribute to improving safeguarding within sport organizations. Selection processes grounded in knowledge and aptitude may serve as an alternative to reforming the existing system, which in many instances is founded on power dynamics.

Therefore, in addition to education, specialized HR expertise may represent an important component of future safeguarding strategies within sports organizations, shaping workplace culture and ensuring security.

The primary point to highlight is that HR may provide practical mechanisms for translating OOP principles into tangible HR tools such as a code of ethics, complaint management procedures, bystander training programs, managerial accountability measures, etc. These initiatives are aimed at fostering a safer environment, thereby contributing to a reduction in future incidents (26).

Beyond the duty of prevention and establishing a secure working environment for athletes, the role of psychologists must also be emphasized. Professionals with expertise in this domain should assume key responsibilities in the rehabilitation process and support for victims of such traumas related to workplace psychological terror within the sports community. In most cases, investigations into recent events in Hungary fall under the jurisdiction of national sports associations. The effectiveness and independence of the committees created by these federations have often been questioned both within the sports community and in public opinion. One possible future policy direction would be to consider establishing an independent panel of experts to investigate and help prevent similar cases within an appropriate supervisory framework body under government oversight. Additionally, this sports safety panel could also offer guidance to sports associations and educators, in collaboration with university researchers in this field, on preventive measures.

Despite the limitations of this preliminary study, the findings suggest that strengthening safeguarding measures and organizational support across different levels of sport may contribute to reducing workplace psychological terror. Because the present study did not directly evaluate the effectiveness of organizational policies or governance structures, these implications should be regarded as recommendations for future research and practice rather than conclusions demonstrated by the data. Future studies should investigate which organizational, leadership, and HR interventions are most effective in preventing workplace psychological terror and promoting safe sporting environments within Hungary. Although our analysis of the measurement instrument is preliminary, the results from the section of the questionnaire examined suggest that this set of questions could serve as a promising starting point for further methodological studies and subsequent validation.

## Data Availability

The original contributions presented in the study are included in the article/Supplementary Material, further inquiries can be directed to the corresponding author.
