# Supplementary material for: Preliminary study on the prevalence of workplace psychological terror among Hungarian athletes
Source: Front Sports Act Living. 2026 Aug 7;8:1828117. doi: 10.3389/fspor.2026.1828117 (PMC13493506; doi:10.3389/fspor.2026.1828117)
Supplement: Supplementary file 1 [file Table1.docx]

APPENDIX A. SUPPLEMENTARY TABLES

**Supplementary Table 1. Prevalence and Sources of Treatment Violating Human Dignity** in Sports Based on the Responses of Hungarian Athletes

| **Question Block** | **Category** | **Count** | **Percent (%)** |
| --- | --- | --- | --- |
| **The survey asked athletes whether they had experienced treatment violating human dignity during their career (e.g., being picked on, mocked, humiliated, ostracized, teased, or having rumors spread about them).** | Athletes who personally experienced it. | 45 | 20 |
|  | Athletes who personally experienced it, and who also reported awareness of other cases. | 74 | 33 |
|  | Athletes who did not experience it themselves but knew one or more cases directly from victims. | 57 | 25 |
|  | Athletes who did not experience it. | 49 | 22 |
| According to the survey, respondents indicated that either they themselves or someone close to them experienced treatment violating human dignity, in the following forms:  (multiple options could be selected) | Mocking, teasing | 106 | 20,8 |
|  | The spreading of malicious rumors | 60 | 11,8 |
|  | Humiliation, shaming in front of others (initiation also fell under this category) | 64 | 12,6 |
|  | Exclusion, marginalization | 67 | 13,2 |
|  | Attacks criticizing the athlete’s private life (e.g., lifestyle, family situation, choice of partner, financial situation, criticism of my private decisions, etc.) | 54 | 10,6 |
|  | Being picked on | 68 | 13,4 |
|  | Fear-mongering, intimidation | 36 | 7,1 |
|  | Being bossed around | 33 | 6,5 |
|  | Harassment due to political, religious, etc. beliefs | 15 | 2,9 |
|  | Other | 6 | 1,2 |
| Treatment violating human dignity was reported by athletes as coming from the following person(s), either in their own case or in a case involving someone close to them.  (multiple options could be selected) | Teammate/sporting partner | 138 | 40,9 |
|  | Coach | 93 | 27,6 |
|  | Sport leader (e.g., club managers, club owners, association managers and responsible employees, etc.) | 27 | 8 |
|  | Private relationship (e.g., boyfriend/girlfriend/partner) | 19 | 5,6 |
|  | Media (e.g., journalist) | 16 | 4,7 |
|  | Parent | 21 | 6,2 |
|  | Competition staff member (e.g., judges, organizers) | 9 | 2,7 |
|  | Other | 8 | 2,4 |
|  | Other team members (e.g., massage therapist, doctor) | 6 | 1,8 |

**Supplementary Table 2. Prevalence and Sources of Verbal Abuse in Sports** Based on the Responses of Hungarian Athletes

| **Question Block** | **Category** | **Count** | **Percent (%)** |
| --- | --- | --- | --- |
| **The survey asked athletes whether they had experienced verbal abuse during their athletic career, including behaviors such as deliberately hurtful shouting, foul language, constant interruptions, suppression of opinions, lack of communication, or being ignored.** | Athletes who personally experienced it. | 63 | 28 |
|  | Athletes who personally experienced it, and who also reported awareness of other cases. | 86 | 38 |
|  | Athletes who did not experience it themselves but knew one or more cases directly from victims. | 26 | 12 |
|  | Athletes who did not experience it. | 50 | 22 |
| The survey captured reports of verbal abuse experienced either personally by athletes or by someone close to them, in the following forms:  (multiple options could be selected) | Intentionally loud and hurtful shouting | 103 | 14,1 |
|  | Condescending, disparaging communication | 85 | 11,6 |
|  | Aggressive or offensive language | 89 | 12,2 |
|  | Constant criticism (insulting or demeaning) | 68 | 9,3 |
|  | Comments that aggressively criticize performance. | 67 | 9,2 |
|  | Sarcastic and cynical hurtful remarks | 67 | 9,2 |
|  | Verbal humiliation in front of others | 60 | 8,2 |
|  | A complete lack of communication (e.g., silent treatment) | 52 | 7,1 |
|  | Excessive criticism of one’s physical appearance | 48 | 6,6 |
|  | Prohibition of expressing my opinion | 39 | 5,3 |
|  | Constant interruptions | 25 | 3,4 |
|  | Verbal threats | 25 | 3,4 |
|  | Other | 4 | 0,5 |
| Athletes reported that they or someone close to them experienced verbal abuse from the following person(s):  (multiple options could be selected) | Coach | 129 | 37,3 |
|  | Teammate/sporting partner | 94 | 27,2 |
|  | Sport leader (e.g., club managers, club owners, association managers and responsible employees, etc.). | 33 | 9,5 |
|  | Parent | 32 | 9,2 |
|  | Private relationship (e.g., boyfriend/girlfriend/partner) | 18 | 5,2 |
|  | Competition staff member (e.g., judges, organizers) | 15 | 4,3 |
|  | Media (e.g., journalist) | 11 | 3,2 |
|  | Other team members (e.g., massage therapist, doctor) | 8 | 2,3 |
|  | Other | 6 | 1,7 |

**Supplementary Table 3. Experiences and Sources of Fear of Punishment in Sports B**ased on the Responses of Hungarian Athletes

| **Question Block** | **Category** | **Count** | **Percent (%)** |
| --- | --- | --- | --- |
| **Athletes were asked whether they had felt fear of potential punishment during their sports career, including situations such as extra training, being sent home, losing sponsorships, or exclusion from the team.** | Athletes who personally felt it. | 66 | 29,33 |
|  | Athletes who personally felt it, and who also reported awareness of other cases. | 58 | 25,78 |
|  | Athletes who did not feel it themselves but knew one or more cases directly from victims. | 19 | 8,44 |
|  | Athletes who did not experience it. | 82 | 36,44 |
| Fear-inducing situations were reported by athletes as being experienced either personally or by someone close to them during their athletic career:  (multiple options could be selected) | Immediately being sent home from a training session | 71 | 18 |
|  | Being left out of the team / Being excluded from the team | 65 | 16,5 |
|  | Intentionally overstrained training sessions as a form of punishment | 59 | 14,9 |
|  | Change in the coach’s attitude/approach | 53 | 13,4 |
|  | Being temporarily benched | 42 | 10,6 |
|  | Suspension from training | 41 | 10,4 |
|  | Financial penalty | 24 | 6,1 |
|  | Loss of sponsorship support, termination of contract | 13 | 3,3 |
|  | Ban from competition | 14 | 3,5 |
|  | Public shaming, negative media coverage | 10 | 2,5 |
|  | Other | 3 | 0,8 |
| Athletes reported that they or someone close to them experienced fear caused by the following person(s):  (multiple options could be selected) | Coach | 131 | 55,5 |
|  | Sport leader (e.g., club managers, club owners, association managers and responsible employees, etc.). | 36 | 15,3 |
|  | Teammate/sporting partner | 29 | 12,3 |
|  | Parent | 16 | 6,8 |
|  | Competition staff member (e.g., judges, organizers) | 8 | 3,4 |
|  | Private relationship (e.g., boyfriend/girlfriend/partner) | 5 | 2,1 |
|  | Media (e.g., journalist) | 5 | 2,1 |
|  | Other team members (e.g., massage therapist, doctor) | 3 | 1,3 |
|  | Other | 3 | 1,3 |

**Supplementary Table 4. Experiences and Sources of Negative Discrimination in Sports** Based on the Responses of Hungarian Athletes

| **Question Block** | **Category** | **Count** | **Percent (%)** |
| --- | --- | --- | --- |
| **The survey asked athletes whether they had experienced negative discrimination during their sports career, such as unreasonably high expectations (e.g., extra tasks) or undervaluation of performance and potential (e.g., lack of training opportunities or being benched).** | Athletes who personally experienced it. | 49 | 21,97 |
|  | Athletes who personally experienced it, and who also reported awareness of other cases. | 56 | 25,11 |
|  | Athletes who did not experience it themselves but knew one or more cases directly from victims. | 22 | 9,87 |
|  | Athletes who did not experience it. | 96 | 43,05 |
| Athletes reported that they or someone close to them experienced negative discrimination during their athletic career for the following reasons:  (multiple options could be selected) | Physical attributes | 70 | 32,4 |
|  | Age | 37 | 17,1 |
|  | Reputation/athletic achievements | 30 | 13,9 |
|  | Family background | 23 | 10,6 |
|  | Economic status | 19 | 8,8 |
|  | Other | 16 | 7,4 |
|  | Race (e.g., nationality, ethnicity, etc.) | 7 | 3,2 |
|  | Political views | 5 | 2,3 |
|  | Gender | 3 | 1,4 |
|  | Religious beliefs | 2 | 0,9 |
|  | Sexual orientation | 4 | 1,9 |
| Athletes reported that they or someone close to them experienced negative discrimination:  (multiple options could be selected) | Coach | 104 | 46 |
|  | Teammate/sporting partner | 54 | 23,9 |
|  | Sport leader (e.g., club managers, club owners, association managers and responsible employees, etc.). | 32 | 14,2 |
|  | Parent | 14 | 6,2 |
|  | Private relationship (e.g., boyfriend/girlfriend/partner) | 7 | 3,1 |
|  | Media (e.g., journalist) | 6 | 2,7 |
|  | Competition staff member (e.g., judges, organizers) | 5 | 2,2 |
|  | Other | 3 | 1,3 |
|  | Other team members (e.g., massage therapist, doctor) | 1 | 0,4 |

**Supplementary Table 5. Experiences and Sources of Positive Discrimination in Sports B**ased on the Responses of Hungarian Athletes

| **Question Block** | **Category** | **Count** | **Percent (%)** |
| --- | --- | --- | --- |
| **The survey asked athletes whether they had experienced positive discrimination during their sports career in the form of special treatment.** | Athletes who personally experienced it. | 71 | 31,98 |
|  | Athletes who personally experienced it, and who also reported awareness of other cases. | 54 | 24,32 |
|  | Athletes who did not experience it themselves but knew one or more cases directly from victims. | 30 | 13,51 |
|  | Athletes who did not experience it. | 67 | 30,18 |
| Positive discrimination or preferential treatment was reported by athletes in cases they were familiar with, arising from one of the following factors.  (multiple options could be selected) | Reputation/athletic achievements | 69 | 27,8 |
|  | Physical attributes | 43 | 17,3 |
|  | Other | 33 | 13,3 |
|  | Age | 25 | 10,1 |
|  | Family background | 30 | 12,1 |
|  | Economic status | 21 | 8,5 |
|  | Gender | 8 | 3,2 |
|  | Religious beliefs | 5 | 2 |
|  | Sexual orientation | 6 | 2,4 |
|  | Political views | 5 | 2 |
|  | Race (e.g., nationality, ethnicity, etc.) | 3 | 1,2 |
| Athletes reported favorable treatment as coming from the following person(s):  (multiple options could be selected) | Coach | 136 | 41,3 |
|  | Teammate/sporting partner | 53 | 16,1 |
|  | Parent | 32 | 9,7 |
|  | Sport leader (e.g., club managers, club owners, association managers and responsible employees, etc.). | 36 | 10,9 |
|  | Private relationship (e.g., boyfriend/girlfriend/partner) | 23 | 7 |
|  | Competition staff member (e.g., judges, organizers) | 18 | 5,5 |
|  | Other team members (e.g., massage therapist, doctor) | 17 | 5,2 |
|  | Media (e.g., journalist) | 10 | 3 |
|  | Other | 4 | 1,2 |

**Supplementary Table 6. Prevalence and Sources of Sexual Harassment in Sports** Based on the Responses of Hungarian Athletes

| **Question Block** | **Category** | **Count** | **Percent (%)** |
| --- | --- | --- | --- |
| **The survey asked athletes whether they had experienced a situation during their athletic career that could be interpreted as sexual harassment.** | Athletes who personally experienced it. | 12 | 5,43% |
|  | Athletes who personally experienced it, and who also reported awareness of other cases. | 11 | 4,98% |
|  | Athletes who did not experience it themselves but knew one or more cases directly from victims. | 18 | 8,14% |
|  | Athletes who did not experience it. | 180 | 81,45% |
| Athletes reported cases they were aware of in which sexual harassment was committed by the following person(s):  (multiple options could be selected) | Coach | 11 | 61,11 |
|  | Teammate/sporting partner | 4 | 22,22 |
|  | Parent | 1 | 5,56 |
|  | Private relationship (e.g., boyfriend/girlfriend/partner) | 2 | 11,11 |
|  | Other team members (e.g., massage therapist, doctor) | 6 | 33,33 |
|  | Sport leader (e.g., club managers, club owners, association managers and responsible employees, etc.). | 3 | 16,67 |
|  | Media (e.g., journalist) | 0 | 0,00 |
|  | Competition staff member (e.g., judges, organizers) | 0 | 0,00 |
|  | Other | 0 | 0,00 |
| Athletes reported that the individuals responsible for the sexual harassment were the following. (multiple options could be selected) | Coach | 17 | 73,91 |
|  | Teammate/sporting partner | 6 | 26,09 |
|  | Parent | 3 | 13,04 |
|  | Private relationship (e.g., boyfriend/girlfriend/partner) | 2 | 8,70 |
|  | Other team members (e.g., massage therapist, doctor) | 3 | 13,04 |
|  | Sport leader (e.g., club managers, club owners, association managers and responsible employees, etc.). | 6 | 26,09 |
|  | Media (e.g., journalist) | 2 | 8,70 |
|  | Competition staff member (e.g., judges, organizers) | 1 | 4,35 |
|  | Other | 2 | 8,70 |

**Supplementary Table 7. Prevalence and Sources of Physical Abuse in Sports B**ased on the Responses of Hungarian Athletes

| **Question Block** | **Category** | **Count** | **Percent (%)** |
| --- | --- | --- | --- |
| **The survey asked athletes whether they had experienced physical abuse during their athletic career, defined as any physical contact they did not consent to and found unpleasant as an athlete.** | Athletes who personally experienced it. | 18 | 8,14% |
|  | Athletes who personally experienced it, and who also reported awareness of other cases. | 28 | 12,67% |
|  | Athletes who did not experience it themselves but knew one or more cases directly from victims. | 26 | 11,76% |
|  | Athletes who did not experience it. | 149 | 67,42% |
| Athletes reported encountering the following forms of physical abuse:  (multiple options could be selected) | Slapping, hitting, beating | 45 | 28,13 |
|  | Pushing, shoving | 29 | 18,13 |
|  | Deliberately excessive training as punishment (unjustified exhaustion) | 33 | 20,63 |
|  | Performing painful or dangerous exercises | 9 | 5,63 |
|  | Rituals/initiations involving physical pain | 12 | 7,50 |
|  | Deliberately harmful actions by fellow athletes on the sports field (e.g., rough tackles, headbutts, punches). | 14 | 8,75 |
|  | Violent clashes between teammates (in locker rooms, outside the sports field) | 12 | 7,50 |
|  | Other | 5 | 3,13 |
| Athletes reported that physical abuse during their athletic career came from the following individuals:  (multiple options could be selected) | Coach | 60 | 50,42 |
|  | Teammate/sporting partner | 34 | 28,57 |
|  | Parent | 8 | 6,72 |
|  | Private relationship (e.g., boyfriend/girlfriend/partner) | 4 | 3,36 |
|  | Other team members (e.g., massage therapist, doctor) | 2 | 1,68 |
|  | Sport leader (e.g., club managers, club owners, association managers and responsible employees, etc.). | 6 | 5,04 |
|  | Media (e.g., journalist) | 1 | 0,84 |
|  | Competition staff member (e.g., judges, organizers) | 2 | 1,68 |
|  | Other | 2 | 1,68 |

**Supplementary Table 8. Experiences and Sources of Coercion into Prohibited Activities in Sports B**ased on the Responses of Hungarian Athletes

| **Question Block** | **Category** | **Count** | **Percent (%)** |
| --- | --- | --- | --- |
| **The survey asked athletes whether they had experienced coercion to engage in prohibited activities during their athletic career (e.g., taking banned substances, sports betting fraud, financial abuse)** | Athletes who personally experienced it. | 3 | 1,36% |
|  | Athletes who personally experienced it, and who also reported awareness of other cases. | 9 | 4,07% |
|  | Athletes who did not experience it themselves but knew one or more cases directly from victims. | 14 | 6,33% |
|  | Athletes who did not experience it. | 195 | 88,24% |
| Athletes reported being pressured to engage in the following prohibited activities.  (multiple options could be selected) | Use of banned substances | 7 | 14,58% |
|  | Influencing the outcome of a match/competition (e.g., intentional loss, match fixing, etc.) | 12 | 25,00% |
|  | Financial abuse, accepting prohibited financial benefits | 5 | 10,42% |
|  | Cheating during a match/competition | 7 | 14,58% |
|  | Sabotaging rivals | 5 | 10,42% |
|  | Technical violations (e.g., unauthorized equipment, etc.) | 7 | 14,58% |
|  | Other | 5 | 10,42% |
| Athletes reported that pressure or coercion to commit the prohibited act came from the following individuals:  (multiple options could be selected) | Coach | 13 | 29,55% |
|  | Teammate/sporting partner | 8 | 18,18% |
|  | Parent | 2 | 4,55% |
|  | Private relationship (e.g., boyfriend/girlfriend/partner) | 4 | 9,09% |
|  | Other team members (e.g., massage therapist, doctor) | 2 | 4,55% |
|  | Sport leader (e.g., club managers, club owners, association managers and responsible employees, etc.). | 8 | 18,18% |
|  | Media (e.g., journalist) | 0 | 0,00% |
|  | Competition staff member (e.g., judges, organizers) | 2 | 4,55% |
|  | Other | 5 | 11,36% |

**Supplementary Table 9. Experiences and Sources of Emotional Manipulation in Sports** Based on the Responses of Hungarian Athletes

| **Question Block** | **Category** | **Count** | **Percent (%)** |
| --- | --- | --- | --- |
| **Athletes were asked if they had experienced emotional manipulation during their sports career (e.g., deliberately lowering self-esteem, withholding attention or love, emotional blackmail, or conditional affection)** | Athletes who personally experienced it. | 37 | 16,74 |
|  | Athletes who personally experienced it, and who also reported awareness of other cases. | 41 | 18,55 |
|  | Athletes who did not experience it themselves but knew one or more cases directly from victims. | 14 | 6,33 |
|  | Athletes who did not experience it. | 129 | 58,37 |
| Athletes reported that they or someone close to them experienced emotional manipulation in the following ways:(multiple options can be selected): | Deliberate influence on one's self-esteem | 54 | 14,79 |
|  | Withdrawal of attention | 30 | 8,22 |
|  | Emotional blackmail (insecurity, guilt-tripping) | 52 | 14,25 |
|  | Veiled threats | 28 | 7,67 |
|  | Withholding motivation and praise | 49 | 13,42 |
|  | Withdrawal of love and emotional give-and-take | 23 | 6,30 |
|  | Conditional love | 17 | 4,66 |
|  | Withholding information (deliberately minimal communication) | 21 | 5,75 |
|  | Passive-aggressive behavior (not openly hostile) | 29 | 7,95 |
|  | Applying unequal expectations | 31 | 8,49 |
|  | Double standards (deceptive communication and tasks) | 19 | 5,21 |
|  | Deliberate isolation | 12 | 3,29 |
|  | Other | 0 | 0,00 |
| Athletes reported that they or someone close to them experienced emotional manipulation directed at them by the following individuals:  (multiple options could be selected) | Coach | 82 | 47,13 |
|  | Teammate/sporting partner | 30 | 17,24 |
|  | Parent | 22 | 12,64 |
|  | Private relationship (e.g., boyfriend/girlfriend/partner) | 6 | 3,45 |
|  | Other team members (e.g., massage therapist, doctor) | 1 | 0,57 |
|  | Sport leader (e.g., club managers, club owners, association managers and responsible employees, etc.). | 25 | 14,37 |
|  | Media (e.g., journalist) | 4 | 2,30 |
|  | Competition staff member (e.g., judges, organizers) | 4 | 2,30 |
|  | Other (You can briefly explain this in the field below, but it is not mandatory to proceed.) | 0 | 0,00 |
